# A Cross-Sectional Study of Primary Biliary Cirrhosis in Japan: Utilization of Clinical Data When Patients Applied to Receive Public Financial Aid

**DOI:** 10.2188/jea.15.24

**Published:** 2005-04-22

**Authors:** Fumio Sakauchi, Mitsuru Mori, Mikio Zeniya, Gotaro Toda

**Affiliations:** 1 Department of Public Health, Sapporo Medical University School of Medicine.; 2 Division of Gastroenterology and Hepatology, Department of Internal Medicine, Jikei University School of Medicine.

**Keywords:** Liver Cirrhosis, Biliary, antimitochondrial antibody (AMA), Cross-Sectional Studies, public financial aid

## Abstract

BACKGROUND: There have not been many reports regarding primary biliary cirrhosis (PBC) in Asia. We conducted a cross-sectional study of PBC in Japan.

METHODS: In fiscal year 1999, 9,761 patients with symptomatic PBC were registered to receive public financial aid from the Ministry of Health, Labour and Welfare of Japan. For our cross-sectional study we chose 5,805 patients whose clinical data had been written between 1999 and 2000, and statistically analyzed the data, including sex, age, major symptoms, and laboratory data.

RESULTS: Our study estimated that the male-to-female ratio was 1:8.0. The median ages of male and female patients were 59 and 60 years, respectively. The major symptoms and physical findings were as follows: pruritus 53.3%, jaundice 11.3%, xanthomas 5.8%, splenomegaly 38.1%, and esophageal varices 19.1%. Antimitochondrial antibody (AMA) was positive in 86.6%, but its positive rate was lower among Japanese patients than among those in western countries. IgM levels were higher among AMA-positive patients than among AMA-negative patients. Regarding Sjögren’s syndrome, rheumatoid arthritis, chronic thyroiditis, and scleroderma, patients had lower frequencies of complicated autoimmune diseases than those in western countries.

CONCLUSIONS: The male-to-female ratio, frequencies by age group, symptoms and physical findings among patients with PBC were consistent with previous reports in Japan and from other countries. However, positivity of AMA and the frequency of complicated autoimmune diseases were lower among patients in Japan than among those in western countries.

Primary biliary cirrhosis (PBC) is a chronic cholestatic disorder characterized by progressive, nonsuppurative inflammation and destruction of small bile ducts, and the presence of antimitochondrial antibodies (AMA) in the sera. PBC is considered to be associated with disturbances in both cellular and humoral immunity, but the etiology is still uncertain. Although PBC has been described in virtually all parts of the world,^1^^,^^2^ most of the epidemiologic data have been derived from Europe,^3^^,^^4^ and there are not so many reports from Asia.

In Japan symptomatic PBC was specified as one of “the intractable diseases” from 1990. Patients with symptomatic PBC who want to receive public financial aid from the Ministry of Health, Labour and Welfare must sign agreements and write applications. Then they are registered and can receive the public financial aid. The recognition of patients with symptomatic PBC is conducted by each prefecture. In 1999, the Ministry of Health, Labour and Welfare permitted the use of clinical data from the recognized patients with symptomatic PBC. Most prefectures, but not all, provided clinical data to the research committee of intractable hepatic diseases. Therefore, the data was also available to the research committee on the epidemiology of intractable diseases.

In the present study, we tried to elucidate the characteristics of patients with PBC in Japan by utilization of the clinical data when they applied to receive public financial aid, and compared the features of the patients in Japan with those in other countries.

## METHODS

In the fiscal year 1999, 9,761 prevalent cases with symptomatic PBC were registered. For our cross-sectional study we used the clinical data of 6,305 patients because not all prefectures provided the data, and chose the cases of 5,805 patients whose clinical data were written between 1999 and 2000; the data from residual cases were not written during the time, for example written in 1998 or before.

Patients whose conditions met one of the criteria below were diagnosed as having PBC following the previous reports in Japan.^5^^-^^7^

1. Chronic non-suppurative destructive cholangitis (CNSDC) is histologically observed and laboratory data do not contradict PBC.
2. AMA is positive. CNSDC is not histologically observed but histological findings are compatible with PBC.
3. Histological examination is not performed, but AMA is positive and clinical findings and course indicate PBC.

For the patients with symptomatic PBC, the following information was collected from the records: sex, date of birth, date of diagnosis, estimated onset time, symptoms and physical findings, complicated autoimmune diseases, laboratory data including serum levels of bilirubin, alkaline phosphatase (ALP), *γ*-glutamyl transpeptidase (*γ*-GTP), total cholesterol (T-chole), IgM, and AMA. We evaluated symptoms and physical findings, laboratory data, and complicated autoimmune diseases according to sex. We also examined the association of IgM levels with the positivity of AMA. In the present study, frequencies of items in the clinical data were analyzed, excluding “unclear” or blank spaces.

Statistical analysis was performed using SPSS^®^ version 10.0 (SPSS Inc.). The chi-squared test was used for comparing the proportions of two groups, and the Mann-Whitney test was used to evaluate differences in clinical variables. P values less than 0.05 were considered to be statistically significant.

## RESULTS

### Age and sex

Table 1 presents the demographic characteristics of the patients. Of 5,805 patients, 646 (11%) were males, and 5,159 (89%) were females, therefore the male-to-female ratio was approximately 1:8.0, showing a much greater frequency in females. The mean and median ages of all patients were 59 and 60 years, respectively. The median age appeared to be somewhat higher in males (62 years) than in females (59 years). The highest frequencies were in the 60s for both sexes (35.1% for males and 34.0% for females, respectively). We tried to estimate the age at the onset of the disease from the records, and found that the median age was 52 years (55 years for males, 52 years for females, respectively).

**Table 1.** Demographic characteristics of the patients with primary biliary cirrhosis.

			both sexes	males	females
No. of patients			5,805	646	5,159

Age (year)	mean ±standard deviation		59±10.7	60±11.8	59±10.5

	median		60	62	59

	relative frequency (%)				
		<19 years	0.1	0.6	0.1
		20-29	0.6	0.6	0.6
		30-39	2.8	3.3	2.8
		40-49	15.8	13.8	16.1
		50-59	30.4	24.1	31.1
		60-69	34.1	35.1	34.0
		70-79	14.3	19.7	13.7
		80+	1.7	2.8	1.6

		total	100	100	100

### Symptoms and physical findings

Pruritis was present in 53.3%, jaundice in 11.3%, xanthomas in 5.8%, splenomegaly in 38.1%, and esophageal varices in 19.1% of all patients (Table 2). Pruritus and jaundice were found significantly more frequently among males than among females.

**Table 2.** Symptoms and physical findings of the patients with primary biliary cirrhosis (%).*

	both sexes	males	females	p value^†^
Pruritus	53.3 (3,014/5,654)	57.5 (358/623)	52.8 (2,656/5,031)	0.03
Jaundice	11.3 (648/5,717)	15.4 (97/631)	10.8 (551/5,086)	<0.001
Xanthomas	5.8 (310/5,339)	6.4 (38/595)	5.7 (272/4,744)	0.58
Splenomegaly	38.1 (2,100/5,508)	37.8 (232/614)	38.2 (1,868/4,894)	0.89
Esophageal varices	19.1 (957/5,012)	17.3 (95/550)	19.3 (862/4,462)	0.27

* : Denominators are not equal because frequencies of items were analyzed, excluding “unclear” or blank spaces.

† : Chi-squared tests for males vs. females.

### Laboratory data

Key laboratory data are summarized in Table 3. Levels of total bilirubin, ALP, and *γ*-GTP were significantly higher among males than among females, whereas levels of T-Chole and IgM were significantly higher among females than among males. AMA was positive in 86.6% of all cases, and its positive rate was significantly higher among males than among females (90.9% for males, 86.1% for females, respectively). Table 4 shows a significant association of IgM levels with the positivity of AMA, i.e., a higher IgM level, and a higher rate of AMA-positive patients (P for trend <0.001).

**Table 3.** Laboratory findings of the patients with primary biliary cirrhosis.

	both sexes				males				females				p value
		
	n	mean	SD	median	n	mean	SD	median	n	mean	SD	median	
Total bilirubin (mg/dL)	5,387	1.1	2.2	0.6	597	1.3	2.1	0.7	4,790	1.1	2.2	0.6	<0.01*
Alkaline phosphatase (ALP: IU/L)	5,655	486.8	702.4	364	643	537.7	595.9	406.5	5,021	480.3	714.6	359	<0.01*
*γ*-glutamyl transpeptidase (*γ*-GTP: IU/L)	5,638	165.1	246.4	86	629	292.1	345.5	178	5,009	149.1	226.0	79	<0.01*
Total cholesterol (mg/dL)	5,458	214.0	230.0	204	601	204.1	77.6	197	4,857	215.3	242.3	205	<0.01*
Immunoglobulin M (IgM: mg/dL)	4,450	444.6	395.2	349.5	477	426.8	316.0	342	3,973	446.7	403.6	350	<0.01*

Antimitochondrial antibody (AMA) positive (%)	86.6 (4,765/5,502)				90.9 (552/607)				86.1 (4,213/4,895)				<0.01^†^

* : Mann-Whitney tests for males vs. females

† : Chi-squared test for males vs. females

SD: standard deviation

**Table 4.** Association between serum IgM level and positivity of antimitochondrial antibody (AMA) among patients with primary biliary cirrhosis.

	Immunoglobulin M (IgM: mg/dL)			

	-299	300-599	600-899	900+
AMA (+)	2,430 (82.4)	1,456 (90.2)	540 (91.8)	332 (96.8)
AMA (-)	520 (17.6)	158 (9.8)	48 (8.2)	11 (3.2)

Parcentages in parentheses

P for trend <0.001

### Complicated autoimmune diseases

Complications such as Sjögren’s syndrome, rheumatoid arthritis, chronic thyroiditis, and scleroderma including CREST syndrome (calcinosis, Raynaud’s phenomenon, esophageal dysmotility, sclerodactyly, and telangiectases) were found in 13.5%, 7.3%, 4.4%, and 2.0% of patients, respectively (Table 5). All these complicated diseases were found significantly more frequently among females than among males.

**Table 5.** Parcentages of patients with primary biliary cirrhosis complicated with selected autoimmune diseases (%).*

	both sexes	males	females	p value^†^
Sjögren’s syndrome	13.5 (683/5,041)	4.5 (25/555)	14.7 (658/4,486)	<0.001
Rheumatoid arthritis	7.3 (390/5,373)	2.5 (15/596)	7.9 (375/4,777)	<0.001
Chronic thyroiditis	4.4 (258/5,805)	0.8 (5/646)	4.9 (253/5,159)	<0.001
Scleroderma	2 (117/5,805)	0.6 (4/646)	2.2 (113/5,159)	0.011

* : Denominators are not equal because frequencies of items were analyzed, excluding “unclear” or blank spaces.

† : Chi-squared tests for males vs. females.

## DISCUSSION

The patients with PBC were characterized by a high proportion of females, especially of middle age. It has been reported that 90% to 95% of patients with PBC are females, with a median age at the time of diagnosis in the early 50s.^8^^,^^9^^,^^10^. In Japan, the Research Committee on the Epidemiology of Intractable Diseases conducted two rounds of nationwide surveys of PBC.^11^^,^^12^ These surveys reported that the male-to-female ratio was 1:8.3-9.1, and that for males the percentage of patients gradually increased from their 40s, reaching the highest frequency age group between 60 and 70 years old, while for females the former increased from their 30s and the latter was between 50 and 60 years old. Regarding the male-to-female ratio and age distributions of the highest frequencies for both sexes, our results approximately agreed with the two reports from the previous nationwide surveys. Inoue et al. also reported that the male-to-female ratio was 1: 7.9, and the peak incidence was in the 50s.^7^

It is still uncertain why females are more susceptible to autoimmune diseases, including PBC, than males. However, a sex-linked genetic influence and hormonal effect for females is one possible explanation.^13^ Females are considered to have the higher absolute number of CD4+ lymphocytes than males, and it is suggested that cytokine secretion is enhanced in the presence of estrogen.^14^ Moreover, androgens have been found to suppress the activity of autoimmune disease such as systemic lupus erythematosus in animal experiments.^15^ Gonadal steroids are likely to play important roles in modulating autoimmune diseases.

Pruritus, jaundice, and xanthomas are usually found in 55%, 10%, and less than 10% of the patients with PBC, respectively.^1^^,^^2^ Our results for pruritus, jaundice and xanthomas were similar to our theses values. The percentage of splenomegaly among patients with PBC is reported to be 15%,^2^ but in the present study splenomegaly was observed in more patients than previously reported. The recent progress of diagnostic imaging techniques such as ultrasonography and computed tomography may be one possible explanation for this. Frequencies of jaundice and biliary enzyme levels were higher among males than among females. Further studies are required to clarify the association between sex and jaundice and biliary enzymes levels.

In Western countries it has been reported that AMA in PBC occurs in 90% to 95% of patients,^8^^,^^16^ but the positivity of AMA was lower (86.6% of total patients). This may be one of the reasons that Japanese patients with PBC have lower frequency of certain types of AMA (e.g. anti-PDC-E2) than Europeans.^13^ More precise laboratory examinations for various types of AMA might resolve this issue.

Lacerda et al. reported that IgM levels were higher in AMA-positive than AMA-negative patients.^17^ Our results also showed an association of a higher IgM level with a higher frequency of AMA-positive patients.

It is reported that Sjögren’s syndrome, rheumatoid arthritis, thyroid diseases, and scleroderma are found in 75%, 10% to 20%, up to 15%, and 10% to 15% of patients with PBC, respectively.^8^ Patients in the present study had lower frequencies of complicated autoimmune diseases than those in western countries. Other studies in Japan have also reported lower complications of these diseases,^5^^,^^6^^,^^7^ but the difference in frequency of complicated autoimmune diseases between Japanese and Western people have not been clearly explained. Polymorphism of the human leukocyte antigen (HLA) might be related to the difference in races.^18^^,^^19^ Our study found a higher frequency of complicated autoimmune diseases among females than among males. This fact also shows that gonadal steroids are likely to play important roles in modulating autoimmune diseases.

In conclusion, using established recorded data, we described the clinical and biochemical features of PBC in Japan. The male-to-female ratio, frequencies by age groups, symptoms and physical findings among patients with PBC were consistent with previous reports in Japan and from other countries. However, positivity of AMA and the frequencies of associated autoimmune diseases were lower among patients in Japan than among those in western countries.
